# Supplementary material for: Comprehensive Annotation and Functional Exploration of MicroRNAs in Lettuce
Source: Front Plant Sci. 2021 Dec 24;12:781836. doi: 10.3389/fpls.2021.781836 (PMC8739914; doi:10.3389/fpls.2021.781836)
Supplement: Supplementary file 2 [file Data_Sheet_8.ZIP › GSM2230749.plot/Lsa-miRN3_Lsat_1_v5_gn_9_122541.1_1065_TPlot.pdf]

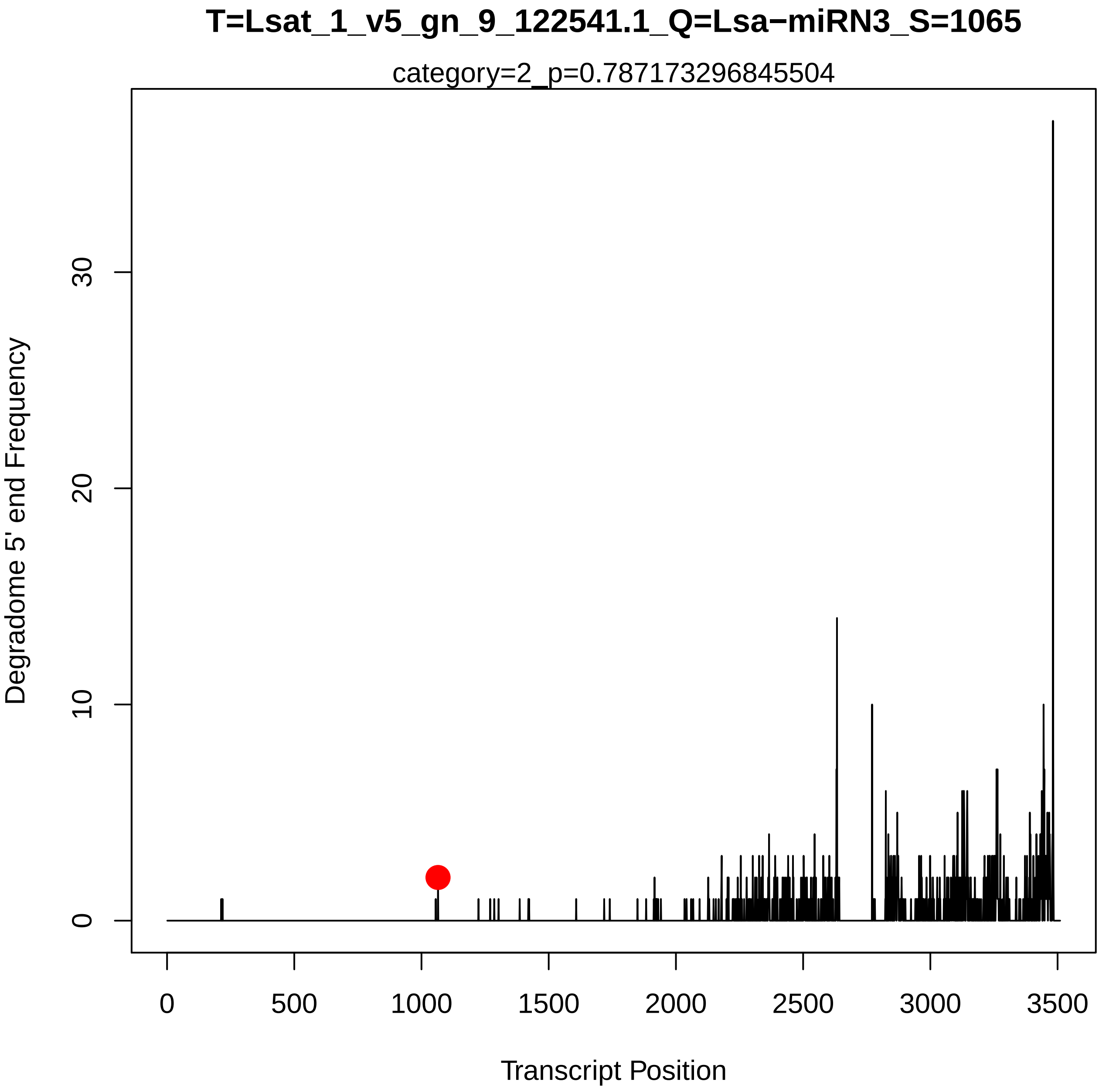
T=Lsat_1_v5_gn_9_122541.1_Q=Lsa−miRN3_S=1065
category=2_p=0.787173296845504
Degradome 5' end Frequency
0
10
20
30
0
500
1000
1500
2000
2500
3000
3500
Transcript Position
